# Pleiotropy between language impairment and broader behavioral disorders—an investigation of both common and rare genetic variants

**DOI:** 10.1186/s11689-021-09403-z

**Published:** 2021-11-13

**Authors:** Ron Nudel, Vivek Appadurai, Alfonso Buil, Merete Nordentoft, Thomas Werge

**Affiliations:** 1 Institute of Biological Psychiatry, Mental Health Centre Sct. Hans, Mental Health Services Copenhagen, Roskilde, Denmark; 2 The Lundbeck Foundation Initiative for Integrative Psychiatric Research, iPSYCH, Aarhus, Denmark; 3 CORE - Copenhagen Research Centre for Mental Health, Mental Health Centre Copenhagen, Copenhagen University Hospital, Copenhagen, Denmark; 4 Department of Clinical Medicine, Faculty of Health and Medical Sciences, University of Copenhagen, Copenhagen, Denmark

**Keywords:** Specific language impairment, Autism spectrum disorder, Attention deficit/hyperactivity disorder, Schizophrenia, Polygenic risk score, Exome sequencing

## Abstract

**Background:**

Language plays a major role in human behavior. For this reason, neurodevelopmental and psychiatric disorders in which linguistic ability is impaired could have a big impact on the individual’s social interaction and general wellbeing. Such disorders tend to have a strong genetic component, but most past studies examined mostly the linguistic overlaps across these disorders; investigations into their genetic overlaps are limited. The aim of this study was to assess the potential genetic overlap between language impairment and broader behavioral disorders employing methods capturing both common and rare genetic variants.

**Methods:**

We employ polygenic risk scores (PRS) trained on specific language impairment (SLI) to evaluate genetic overlap across several disorders in a large case-cohort sample comprising ~13,000 autism spectrum disorder (ASD) cases, including cases of childhood autism and Asperger’s syndrome, ~15,000 attention deficit/hyperactivity disorder (ADHD) cases, ~3000 schizophrenia cases, and ~21,000 population controls. We also examine rare variants in SLI/language-related genes in a subset of the sample that was exome-sequenced using the SKAT-O method.

**Results:**

We find that there is little evidence for genetic overlap between SLI and ADHD, schizophrenia, and ASD, the latter being in line with results of linguistic analyses in past studies. However, we observe a small, significant genetic overlap between SLI and childhood autism specifically, which we do not observe for SLI and Asperger’s syndrome. Moreover, we observe that childhood autism cases have significantly higher SLI-trained PRS compared to Asperger’s syndrome cases; these results correspond well to the linguistic profiles of both disorders. Our rare variant analyses provide suggestive evidence of association for specific genes with ASD, childhood autism, and schizophrenia.

**Conclusions:**

Our study provides, for the first time, to our knowledge, genetic evidence for ASD subtypes based on risk variants for language impairment.

**Supplementary Information:**

The online version contains supplementary material available at 10.1186/s11689-021-09403-z.

## Background

One of the most fundamental aspects of human behavior is communication through language. At the same time, it is also one of the most remarkable ones; children can acquire their mother tongue with ease and without conscious effort, and, yet, the mechanisms underlying this ability are largely unknown. There are many theories as to the nature and structure of human language, and they differ from one another from both the organizational and the representational perspectives. This, in turn, may have implications for accounts of language acquisition which use frameworks and concepts anchored in those theories [1]. However, from the molecular or genetic point of view, we do not need to presuppose much about the linguistic nature of the mechanisms which allow the child to acquire language or their relation to other cognitive domains. All the same, using genetics to investigate them may help answer some questions which pertain to higher levels of linguistic ability. Furthermore, it may also help answer questions which pertain to the links between linguistic ability and other behavioral or even physiological traits, in much the same way in which links between other behavioral and physiological traits and disorders have been found [2]. Thus, by exploring the genetic relationship between a primary form of language impairment and broader behavioral phenotypes, we could potentially identify pathways that may affect both language and other traits, which could, in turn, inform theories of language acquisition and development as well.

It has long been known, from twin studies and other family-based studies, that language ability and some language disorders are heritable [3]. For developmental spoken language disorders, pooling together twin data from across several studies obtained overall concordance rates of 83.6% for monozygotic twins and 50.2% for dizygotic twins, indicating a strong genetic component [3]. Twenty years ago, *FOXP2* became the first gene implicated in a speech and language disorder [4]. While the disorder was multi-faceted in terms of its behavioral phenotype, its genetic cause was a point mutation in a single gene. However, there are other disorders in which language is or may be impaired, and these may be complex, meaning that several genetic and environmental factors may combine to confer an increased risk of having them. Some complex disorders involve a child’s broad behavioral neurodevelopment, and they include, among others, autism spectrum disorder (ASD) and attention deficit/hyperactivity disorder (ADHD), both of which are heritable [5] and may involve language deficits [6, 7]. In contrast, another disorder, namely, specific language impairment (SLI), is diagnosed when linguistic development is below age expectation in an otherwise typically developing child [8]. In recent years, the diagnostic criteria have changed, implementing a shift towards less exclusionary criteria and resulting in a new label: developmental language disorder^1^, although ASD remained an exclusionary criterion [9]. Like ASD and ADHD, SLI is a complex disorder [10]. In contrast to the aforementioned monogenic speech and language disorder, *FOXP2* was not found to play a major role in SLI susceptibility, thus suggesting a different genetic architecture and, perhaps, a more complex one [11]. Interestingly, several linguistic domains may also be impaired in schizophrenia [12], a psychiatric disorder not typically diagnosed in children, and language deficits in schizophrenia show familial aggregation [13]. Some studies implicated *FOXP2* either in either schizophrenia itself [14] or in language ability in schizophrenia patients [15], whereas other studies found no such associations [16, 17]. Of note, the SNP-based heritabilities of ASD, ADHD, and schizophrenia were recently estimated to be ~10%, ~20%, and ~13%, respectively, in the iPSYCH sample (which was also used in this study) [18].

Especially in the case of SLI and autism (autism being part of and arguably the core disorder within ASD), the (perhaps, superficial) similarities in linguistic impairment led to the question being raised of whether SLI and autism were on one continuum [19]. Until recently, most studies trying to answer this question focused on the linguistic deficits in SLI and autism, with some reporting similar linguistic domains being impaired in both disorders, and others reporting that children with SLI and children with autism are impaired on different domains [19–23], but it is generally said that the core deficits in SLI involve spoken language production and comprehension and the domains commonly affected in SLI are “structural” (phonology, morphology syntax and semantics), whereas the core linguistic deficit in autism affects mostly pragmatics (language use and hence social communication), although some children with SLI may exhibit some overlaps in the affected domains with children with autism, and vice versa [24]. What further complicated things was that some genes were linked to both disorders; for example, *CNTNAP2* (itself a *FOXP2* target [25]) was one of those genes [25, 26] (of note, it was also linked to schizophrenia [27]). Moreover, the top associations in the first genome-wide association study (GWAS) of SLI, in the model for child genetic effects, were with variants in genes previously implicated in ASD (and even in ADHD and schizophrenia) [28]; these included *CNTN5* [29], *RBFOX3* [30], and *THRB* [31, 32] (see Supplementary Table S1 for the top 10 SNPs from the discovery analysis from this study, i.e., with the updated dataset as per below, and corresponding genes). It was suggested that such observations (both linguistic and genetic) could be explained by a model incorporating genetic interactions, rather than only additive genetic overlaps; this type of model could account for genetic overlaps, while maintaining distinct linguistic profiles for SLI and autism [33]. It is worth mentioning that the reported genetic overlaps concerning specific genes were not always of the same nature across disorders; for example, common variants in *CNTNAP2* were associated with a language trait in children with SLI [25], but for ASD, a rare variant in that gene was also reported [34]. However, this is in line with theoretical accounts of disease-causing genes and also with experimental data showing associations with common variants in genes which are involved in related monogenic diseases [35]. This implies that both common and rare variants, the latter possibly having stronger deleterious effects, should ideally be examined when assessing genetic overlaps between disorders.

### Polygenic risk scores as a tool for investigating cross-disorder genetic overlaps

A polygenic risk score (PRS) is an aggregate score that reflects an individual’s genetic predisposition to a trait or a disease, as estimated based on prior genetic association data (typically from a GWAS for the given trait or disease). A PRS trained on a sample comprising cases and controls for a given disease is often used as a predictor for the same disease in an independent sample, but a PRS trained on one disease can be used to try to predict the risk of having another disease. This is known as a cross-disorder analysis and has been done for several psychiatric disorders; it provides a way of assessing genetic overlaps across disorders [36].

### Polygenic risk scores in the clinical setting

Since their first use in a study of human disease [37], PRSs have become a popular tool in research. The nature of the PRS, i.e., being one aggregate score capturing an individual’s genetic predisposition to having a particular trait, also means it can be readily used by researchers in disciplines typically far removed from genetics, such as the social sciences, where it can be incorporated into statistical models, thus allowing the integration of genetic information and social outcomes [38]. But although PRSs have been successfully used in research contexts, typically allowing to differentiate cases and controls at the group level, they cannot, as of now, be used as predictors for disease at an individual level [39]. Nonetheless, integrating PRSs into the clinical setting remains one of the main goals of PRS research, and, even though a PRS cannot stratify individuals from the entire population based on the individual probabilities of their developing a disease, it could, together with clinical risk factors, potentially help identify a group of individuals with a particularly high risk for some diseases [40]. One example of a successful application of this approach was using a PRS to identify individuals at high risk of coronary artery disease; using a PRS, the authors were able to identify a group of individuals (8% of the population) with high risk of developing coronary artery disease, with an odds ratio ≥ 3 [41].

### Polygenic risk scores in studies of language-related traits

In psychiatry, where the clinical presentation of various conditions might be more complex, PRSs could be used for distinguishing subtypes of psychiatric disorders. A PRS for schizophrenia has been shown to differentiate schizoaffective bipolar disorder cases from the rest of the bipolar disorder sample in one study [42]. However, when it comes to prognostic value, a recent study found no significant improvement in using a PRS for schizophrenia when predicting poor outcomes (proxies for a poor clinical trajectory, including: aggressive behavior, requiring in-patient psychiatric treatment, prescription of two or more unique antipsychotics, prescription of clozapine, self-harm and homelessness), relative to current standards of care [43], even for models in which the PRS was significantly associated with the proxy (i.e., it significantly explained some of the variance in those traits), which was the case for the first two out of the above six outcomes. This example illustrates the fact that, even if the PRS is significantly associated with an outcome, it does not mean that adding it to the prediction model would improve the performance of the model relative to including only clinical features.

As in the general case of psychiatric disorders described above, studies of language-related disorders are also plagued by the heterogeneity of the disorders, which, in turn, may also influence clinical diagnosis and treatment, even in terms of access to support services in the first place [44]. In this respect, studying the genetic architecture of the disorder could be informative as to the boundaries (and similarities) between disorders with overlapping phenotypes. Studies applying PRSs to language-related traits or disorders in the clinical context are scarce, but attempts have been made to investigate the potential use of PRSs in these settings. A PRS based on several language measures was shown to explain a small proportion of the variance in language and psychosocial problems in 8-year-old children, although this PRS was not genome-wide and consisted of markers in preselected candidate genes [45]. Another recent study examined the potential application of a genome-wide PRS for educational attainment to identifying children with language and literacy problems at an early developmental stage. The PRS for educational attainment significantly explained a small proportion of the variance of language and literacy at age 12, but its predictive ability was overall low and deemed not useful in the clinical setting [46]. In the neighboring field of speech and voice disorders, the use of genetic information in the clinical setting has also been advocated [47]. In addition to investigating the direct association between PRS for a relevant trait and developmental outcomes, clinical practice may also benefit, albeit not immediately, from studies into the genetic overlaps across language-related disorders and traits. For example, previous investigations which used PRSs in a cross-disorder setting identified genetic overlaps between ADHD and reading-related traits (showing a negative association) [48]. Interestingly, these traits also showed a positive association with PRSs for educational attainment in the same study.

In our own previous study (hereafter referred to as the pilot study), which included a family-based cohort comprising children assessed for language, intelligence, and other behavioral traits through test batteries and interviews, we used a PRS trained on the SLI GWAS in trying to predict risk of ASD and ADHD, using case-control datasets for these disorders from among the unrelated children of the cohort (*N* = 391). In our study, we observed that, overall, the PRS significantly predicted some risk of SLI, used as a positive control in the target sample, but it did not predict risk of ASD (mutually exclusive with SLI) or ADHD—or height, used as a negative control [49]. Thus, at least when it came to common variants, we did not observe a genetic overlap between SLI and ASD, or between SLI and ADHD, as captured by a genome-wide PRS. The biggest limitations of the previous study were the sample size and the fact that the ASD diagnosis encompassed children with varying language profiles (as determined from their performance on a receptive language test). The aim of this extended study is thus fourfold: (i) to apply SLI-trained PRS to a much larger sample, which comprises more than ten thousand cases each of ASD and ADHD; (ii) to see whether different results are obtained when examining childhood autism and Asperger’s syndrome (which differ in their linguistic profiles, in this case, based on International Classification of Diseases (ICD) criteria) separately, and to assess whether this could potentially be used to guide clinical diagnosis; (iii) to extend the analysis to include schizophrenia; (iv) to examine potential genetic overlaps between SLI and the other disorders using exome sequencing data which include rare variants in SLI candidate genes and other language-related genes.

## Methods

### Study population and phenotypes

The individuals in this study are part of the Danish iPSYCH case-cohort sample [50], which comprises individuals selected either for having at least one of six disorders (ASD, ADHD, schizophrenia, bipolar disorder, depression and anorexia) or as part of a random population sample. The iPSYCH samples underwent extensive quality control (QC) procedures based on both genetic data and registry data to remove ancestry outliers, duplicate samples, individuals with cryptic relatedness, and individuals with low-quality genotype measures, as described in an earlier study [18]. This resulted in a sample of 65,534 unrelated Danish individuals, as used in previous studies [51–53]. The phenotypes used in this study include the 2016 dataset of diagnoses from the Danish Psychiatric Central Research Register for these 65,534 individuals. The diagnoses correspond to the following ICD-10 [54] codes: ASD (F84.0, F84.1, F84.5, F84.8, and F84.9), childhood autism (F84.0), Asperger’s syndrome (F84.5), ADHD (F90.0), and schizophrenia (F20). Equivalent ICD-8 [55] codes might have been used for schizophrenia (295.x9 excluding 295.79) and childhood autism (299.00), depending on when the individual received the diagnosis. For each phenotype, cases were defined as having the respective diagnosis as per the above codes, and controls were defined as (i) not having the diagnosis in question and (ii) having been included in iPSYCH as part of the random population sample, i.e., an individual who is included only in the case subset of iPSYCH will not be included as a control for another case diagnosis (which they do not have), but they are considered a case for the diagnosis they do have. Individuals in iPSYCH may be cases for more than one disorder.

### Genetic quality control

The samples were genotyped on the Illumina PsychArray v1.0. Preliminary QC steps on the raw genotype data (based on call rates and the Gentrain score) are described in the original iPSYCH paper [50] and subsequent QC is described in a later iPSYCH study [56]. The marker dataset used in this study was filtered further with PLINK [57] v1.90b3o to remove markers with rare variants and non-autosomal markers, and later with v1.90b3.34 to remove one marker from every pair of markers with duplicate positions. The final dataset had markers with a minimum minor allele frequency (MAF) of 0.009632 and maximum missingness of 0.01257. All but 218 markers had Hardy-Weinberg equilibrium *p* value > 1×10^−6^ in controls. We report these numbers and not thresholds, as the QC steps were performed in a larger subset of the iPSYCH sample (a homogeneous sample of European ancestry) than that used in this study, which was selected for the purpose of QCing the markers prior to imputation for another study. Further details are given in that study [18] and in a subsequent study [58]. In total, 242,077 markers were retained following these steps, and 239,582 markers remained after removing markers from the major histocompatibility complex (MHC) region. Note that the above QC describes the marker QC; the final sample used in this study comprised only individuals passing the sample QC as mentioned in the previous section and as described in [18].

### Polygenic risk scores and regression models

The summary statistics used in the construction of the PRS were taken from an updated analysis of a previous SLI GWAS [28]. To our knowledge, this is currently the only GWAS of SLI, and it is based on the largest available sample of SLI families, the SLI Consortium sample. The dataset used in this study was the most strictly QCed one (termed “Correction 1”), as used in our pilot study, a recent study of PRS in SLI, ASD, and ADHD, which details the complete protocol for this dataset [49]. In short, the SLI phenotype in the discovery study was based on proband status and/or low receptive or expressive language scores from a standardized test. The SLI Consortium also employed exclusion criteria which included low non-verbal intelligence and/or an indication of autism, as detailed in the original papers [28, 59–61]. The average numbers of family subsets per single-nucleotide polymorphism (SNP) in the updated GWAS were as follows: 150 case-parents trios, 55 case-mother duos, 12 case-father duos, and 19 cases (and sometimes case parents, but these were few, on average < 1 per SNP). These subsets were generated per marker by the PREMIM tool, which generates the input to EMIM (the software with which the GWAS was performed) [62], in a way that prioritizes case-parents trios. For example, if for a given marker and for a given family both parents and a case have genotypes, then this would be a trio subset. If the paternal genotype is missing for this marker, then this would be a case-mother duo, and so on. The GWAS was family-based (not case-control; only case subsets were used as per the above), whereby the effect estimated for each SNP in the model used in the GWAS was one effect or risk parameter, R_1_ (defined as the factor by which a child’s disease risk is multiplied if they possess one risk allele), so that the increase in risk from carrying two risk alleles was defined as the square of R_1_ [63]. By default, the minor allele was defined as the “risk” allele, but it could also be protective, in which case the effect parameter would be < 1. These effects were used in the calculation of the PRS, similar to the use of odds ratios (ORs). SNPs which had a “warning” value of 1 from EMIM (i.e., there was some problem in the models for those SNPs) were removed from the summary statistics. Further information about the model employed in the discovery GWAS can be found in the supplementary notes for this paper. PRSs were generated for iPSYCH individuals using PRSice v2.2.6 [64] with the following clumping parameters: *r*^2^ value of 0.2 in a 500-kbp window, as recommended for psychiatric traits [36], and a *p* value threshold of 1, both to conform to the protocol used in the pilot study and to increase the accuracy of the PRS (as it has been observed that, when the discovery sample is not very large, including all SNPs can lead to better performance, and both experimental and simulation studies reported better performance when including all SNPs in most cases; this is particularly applicable to cases in which the original GWAS did not identify many genome-wide significant associations) [36, 49, 65, 66]. As before, SNPs from the MHC region and ambiguous (A/T and G/C) SNPs were excluded. For binary traits, the program performs a logistic regression of the phenotype on the PRS and outputs Nagelkerke’s *R*^2^ as well as an adjusted *R*^2^ (the adjustment is for the proportion of cases and prevalence for each phenotype) [67]; to that end, the following prevalence values were provided to PRSice for the ASD, ADHD, and schizophrenia phenotypes, respectively: 1% [68], 5% [69], and 0.4% [70]. In the analyses for the ASD subtypes, the prevalence value used for childhood autism was 0.4% [68], and for Asperger’s syndrome, it was 0.3% [71]. Otherwise, the default parameters of PRSice were used. The logistic regressions of the phenotype on the PRS were also repeated in R v3.3.1 [72] using PRS scaled across the entire sample (using the *scale* function in R with the default parameters), so that the regression odds ratios are derived from coefficients corresponding to a change of 1 standard deviation (SD) in the PRS, as presented in the “Results” section. The reported two-sided *p* values for the models are for these coefficients’ being different from zero, as evaluated by the function in R (using the *t*-distribution for a linear regression (using the *t*-statistic), e.g., for height in the pilot study, and the normal distribution for a logistic regression (using the Wald *z*-score), e.g., for ASD, as implemented in the *lm* and *glm* functions in R, respectively). Confidence intervals (CIs) for the coefficients were estimated using the *confint* function. The sample sizes (cases; controls) for the PRS analyses were as follows: ASD (12,884; 21,321), childhood autism (3,313; 21,634), Asperger’s syndrome (4,710; 21,567), ADHD (15,060; 21,265), schizophrenia (2,867; 21,596).

### Candidate genes for language disorders and traits and rare variant group tests

Since only a very small proportion of the SLI Consortium proband sample was exome-sequenced [73], and given the reported genetic overlaps between monogenic disorders and common variants in phenotypically related disorders, as discussed earlier, we included candidate genes implicated through both common and rare variants in the exome-sequencing analyses. As a first step, we used recent review articles about the genetics of language disorders and related conditions [74, 75], as well as a survey of some of the literature from our recent work on receptive language [76], to identify studies in which at least one of the investigated phenotypes was spoken language impairment or a spoken language trait. We then included two categories of genes in the rare variant analyses: (i) genes implicated directly in spoken language impairment: *CNTNAP2* [25], *CMIP*, and *ATP2C2* [77], *NOP9* [28], *NFXL1* [78], *SETBP1* [79], *NDST4* [80], and *OXR1*, *MUC6*, *SCN9A*, *FAT3*, *KMT2D*, and *PALB2* [73]; (ii) genes implicated in studies of spoken language traits in a general population sample not selected for having low language ability: *ABCC13* [81] and *RORB* [82]. Human leukocyte antigen (HLA) genes were not included due to the complex genetic architecture of the MHC region and the fact that they (and their overlaps across disorders) had already been extensively examined in past studies of SLI, ASD, ADHD, and schizophrenia [52, 83–87]. While additional genes have been implicated in broader disorders or phenotypes involving language, we chose to keep genes reported specifically for spoken language impairment or spoken language traits not in combination with other traits (e.g., not language impairment and reading impairment modeled simultaneously or speech-related disorders, and so on). Additionally, we required that the gene be implicated directly, that is, through a gene-based analysis, or, in case of an association study, that the associated markers be within the gene. This was done to ensure that the rare variant analyses are closer to the PRS analyses (which were based on common variants for SLI, i.e., spoken language impairment)—even though the two approaches differ in methodology and interpretation—and in order to be able to draw conclusions regarding the potential overlaps between spoken language disorders/traits proper and the other phenotypes. Genes were selected from the above studies based on reported significance levels within each study, or on the gene being the top candidate in a given study based on its *p* value or qualitative measures, e.g., genes with co-segregating rare variants or genes highlighted through compound heterozygous inheritance in the exome-sequencing study of SLI. A flowchart summarizing the selection process can be found in Supplementary Figure S1. The starting point for the rare variant analyses was a dataset generated for, and described in detail in, a recent iPSYCH study [88]. The genetic data (exome variants) for this dataset were generated independently of the genotype array data described earlier in the “Methods”; however, individuals failing the iPSYCH sample QC, e.g., on account of having non-Danish ancestry, were excluded from the pedigree/phenotype file provided to the program which performed the tests, so that every individual in the exome-sequencing dataset must also have passed the general iPSYCH sample QC as referenced above. The genomic coordinates for the above list of genes were obtained from Release 19 (GRCh37.p13) of GENCODE, and variants in those positions were extracted from the iPSYCH VCF file using BCFtools v1.9 [89]. The new VCF file was annotated using snpEff v4.3t [90] with the GRCh37.p13 database. The variants kept for downstream analysis were of the following types: frameshift variants; missense variants; nonsense (stop gained) variants; splice site donor, acceptor, or region variants, all with a maximum MAF of 1%. The statistical test employed was the optimized SKAT test (SKAT-O) [91] as implemented in EPACTS v3.2.6 (with the default parameters, apart from the maximum MAF as per the above) [92], for which the variants in each gene were grouped together. SKAT-O optimally combines the burden test (which collapses the variant counts for all markers in a region) and the SKAT (which sums up the squares of the variant score statistics for all markers in a region), both of which examine aggregate variant effects, but perform optimally in different scenarios; the burden test is most suitable when most variants in a region are causal and their effects are in the same direction, and SKAT is most suitable when a large proportion of the variants in a region are either non-causal, or have effects that are in different directions [91]. The *p* value for the test is for the enrichment of rare variant associations per gene, and the ratio Rho reflects the optimal combination of the two kinds of tests (1 corresponds to a pure burden test, and 0 corresponds to a pure SKAT). No variants (passing QC) were found in *CNTNAP2*, and the gene was therefore not included in the tests. The sample sizes (cases; controls) for the rare variant analyses were as follows: ASD (9,579; 8,782), childhood autism (2,343; 8,987), Asperger’s syndrome (3,482; 8,944), ADHD (7,396; 8,816), schizophrenia (1,980; 8,968).

### Comparison with the pilot study

We present results from analyses which used the pilot study [49] sample (run with PRSice v2.2.3) for comparison with the current study, as these results and their contrast or similarity with the results from the iPSYCH sample are important for the interpretation of the findings from the present study. The pilot study sample consisted of unrelated children who were part of a family-based study, the Danish High Risk and Resilience Study – VIA 7, who were assessed for language performance, intelligence, and other behavioral traits [93], as detailed in our previous publications which used the genetic data for these children [49, 76]. The pilot study paper details the sample size and the criteria for the affection status or measurement for each phenotype included here (note that SLI was also termed "narrow language phenotype" in that paper). We include updated and slightly different results here as compared to the pilot study as published, as subsequent QC in the family-based sample revealed some Mendelian errors in child-parent duos not previously identified (as duos are not checked by default by PLINK), as reported in a subsequent study which used the same sample [76]. This did not result in the removal of any duos that were not removed at a later stage anyway (in the relatedness check), but a number of markers and genotypes were removed (the conclusions of the original study were not affected by this). For the binary traits in the pilot study, the regression models were the same as those described for iPSYCH, with the same prevalences as mentioned earlier for ASD and ADHD, and a prevalence of 7% for SLI [94]. For height, a linear regression was performed with covariates for the age at measurement and sex. The reported *R*^2^ for the PRS was calculated as the *R*^2^ for the full model (height regressed on PRS and covariates) minus the *R*^2^ for the null model (height regressed on the covariates), as implemented in PRSice. Lastly, we report some new analyses which used the pilot study sample but were not included in the pilot study, as they are relevant for comparison with the iPSYCH sample.

### Difference in PRS between childhood autism cases and Asperger’s syndrome cases

Following the regression analyses and in order to evaluate the difference in PRS between childhood autism and Asperger’s syndrome, we performed a Mann-Whitney *U* test with the *wilcox.test* function in R, using the scaled PRS. We performed a one-sided test, as we expected cases of childhood autism to have a higher PRS than cases of Asperger’s syndrome. For this purpose, we excluded a small number of children who had both diagnoses (*N* = 175). Area under the curve (AUC) values were computed with the *auc* function of the pROC package v1.17.0.1 in R [95]. For the purpose of calculating the AUC, childhood autism cases were defined as “cases” (affection status 1) and Asperger’s syndrome cases were defined as “controls” (affection status 0).

## Results

The results of all PRS analyses are shown in Table 1. Overall, the SLI-trained PRS, which was previously found to be predictive of SLI in an independent sample in our pilot study, was not predictive of the risk of ASD or ADHD (adjusted *R*^2^ close to 0%, neither of them significant after Bonferroni correction, *N* = 5), in line with and thus replicating the results of our pilot study; for the additional phenotype of schizophrenia, the PRS was not predictive. While the result for ADHD was similar in terms of effect size, *R*^2^ and *p* value in both studies, the result for ASD was not. The explanatory power of the PRS was close to zero in both cases (~0.02% in the pilot study; ~0.01% in the current study), but the association in the pilot study was in the opposite direction compared to the new result for ASD, and the latter was at least nominally significant, unlike the former result. Since the current sample is much larger, the effect estimate is more accurate, and the confidence interval is smaller. This new result suggests that, while, by large, the genetic overlap (from common variants) between SLI and ASD is small, it may nonetheless be different from zero, and that at least some of the overlapping loci have effects in the same direction. This is illustrated more strongly when comparing the models for childhood autism and Asperger’s syndrome: even though there were fewer cases of childhood autism compared to both ASD in general and Asperger’s syndrome in particular, the model for childhood autism performed better, with *R*^2^ ≈ 0.04% and *P* = 0.001, which survives Bonferroni correction for multiple testing (*N* = 5), whereas the model for Asperger’s syndrome was not predictive. The above models tested each ASD subtype against controls; we therefore sought to evaluate the difference between the two case groups directly. Our Mann-Whitney *U* test found a significant difference between the childhood autism and Asperger’s syndrome case groups (*W* = 7,353,100, difference in location = 0.059, *P* = 0.006, lower bound of a 95% confidence interval (CI) = 0.02). This corresponds to an AUC of ~52%, which is only ~2% over what is considered completely uninformative. Of note, using the same approach with the updated dataset from the pilot study, we obtain an AUC of ~63% for SLI cases *versus* SLI controls (one-sided *P* = 0.025 for the *U* test), and a similar AUC of ~63% for SLI cases *versus* ASD cases (*P* = 0.09). These results are summarized in Table 2.

**Table 1:** Results of the PRS regression analyses in iPSYCH; updated results from the pilot study sample are shown for comparison

Phenotype	*R*^2^/Nagelkerke’s *R*^2^ (%)	Adjusted *R*^2^ (%)	Scaled PRS beta/ odds ratio (95% CI)	*P* value
iPSYCH sample^*^				
*ASD*	*0.017*	*0.007*	*1.02 (1.00–1.05)*	*0.037*
**Childhood autism**	**0.079**	**0.042**	**1.06 (1.02***–***1.10)**	**0.001**
Asperger’s syndrome	0.002	0.001	1.01 (0.98*–*1.04)	0.552
ADHD	0.002	0.002	1.01 (0.99*–*1.03)	0.426
Schizophrenia	0.002	0.001	0.99 (0.95*–*1.03)	0.613
Pilot study sample^**^				
ASD	0.017	0.017	0.97 (0.59*–*1.57)	0.887
ADHD	0.008	0.009	1.02 (0.75*–*1.39)	0.897
SLI	3.5	5.09	1.60 (1.02*–*2.54)	0.041
Height	0.006	NA	0.07 ((− 0.98) *–*1.13)	0.895

The adjusted *R*^2^ includes an adjustment for the prevalence and proportion of cases in each sample, as implemented in the PRSice software. Associations in boldface survive Bonferroni correction for multiple testing (not applicable to positive or negative controls). Associations in italic are only nominally significant (not applicable to positive or negative controls)

*PRS* polygenic risk score, *CI* confidence interval, *ASD* autism spectrum disorder, *ADHD* attention deficit/hyperactivity disorder, *SLI* specific language impairment, *NA* not applicable

The *R*^2^ reported for height is a true *R*^2^ from a linear regression and not a Nagelkerke’s *R*^2^, and the effect is the beta (regression coefficient) and not an odds ratio; it should therefore be judged relative to 0 and not to 1. The height variable was collected in different ways at the Copenhagen and Aarhus centers for the VIA 7 study; the Aarhus center used, for the most part, a uniform method of measuring height with a special device, but we note that the PRS is not significantly predictive of height even if we restrict the analysis to include only children measured in Aarhus. We include this for the sake of completeness, even though the sample size is much smaller and thus the estimates are less reliable (*R*^2^ = 0.7%, *P* = 0.419)

*72,932 SNPs were included in the PRS

**80,121 SNPs were included in the PRS

**Table 2:** Results of group comparisons using the Mann-Whitney *U* test or AUC analyses

Group 1	Group 2	Type of analysis	Result	*P* value
**Childhood autism cases (iPSYCH sample)**	**Asperger’s syndrome cases (iPSYCH sample)**	**Mann-Whitney** ***U*** **test**	***W*** **= 7,353,100; difference in location = 0.059**	**0.006**
Childhood autism cases (iPSYCH sample)	Asperger’s syndrome cases (iPSYCH sample)	AUC	0.5167	NA
SLI cases (pilot study sample)	SLI controls (pilot study sample)	AUC	0.6276	NA
SLI cases (pilot study sample)	ASD cases (pilot study sample)	AUC	0.6303	NA

Associations in bold survive Bonferroni correction for multiple testing

*AUC* area under the curve, *NA* not applicable, *ASD* autism spectrum disorder, *SLI* specific language impairment

The results of the rare variant analyses are shown in Table 3. Tests for three genes obtained nominally significant *p* values: *NDST4* in ASD, *RORB* in childhood autism, and *SETBP1* in schizophrenia. However, none of these survive Bonferroni correction for multiple testing (*N* = 70).

**Table 3:** Results of the rare variant analyses

Phenotype	ASD			Childhood autism			Asperger’s syndrome			ADHD			Schizophrenia		
Gene	Number of variants	*P* value	Rho	Number of variants	*P* value	Rho	Number of variants	*P* value	Rho	Number of variants	*P* value	Rho	Number of variants	*P* value	Rho
*NOP9*	61	0.059	0	46	0.103	0	49	0.108	0.5	57	0.242	0	47	0.111	0.4
*KMT2D*	454	0.227	0	361	0.545	0	361	0.319	1	435	0.533	1	337	0.126	0.7
*RORB*	14	0.243	1	*11*	*0.033*	*1*	12	0.894	1	14	0.300	1	11	0.397	1
*ABCC13*	1	0.138	NA	1	0.180	NA	1	0.584	NA	1	0.383	NA	1	0.079	NA
*NDST4*	*45*	*0.011*	*0.4*	42	0.270	1	43	0.117	0.4	48	0.215	1	44	0.142	1
*FAT3*	361	0.361	1	289	0.549	1	291	0.420	1	337	0.144	0.6	278	0.726	0
*CMIP*	22	0.402	1	18	1.000	1	18	0.637	0	23	1.000	0	17	0.715	0
*PALB2*	83	0.551	0	64	0.592	0	67	0.827	1	75	0.747	1	65	0.633	0
*SCN9A*	130	0.093	0	103	0.578	0	109	0.396	0	126	0.153	0	102	0.308	1
*MUC6*	31	0.857	1	27	0.816	0	28	1.000	1	32	0.752	1	28	0.815	1
*NFXL1*	26	0.519	1	13	0.695	0	15	0.235	1	17	0.849	1	13	0.559	0
*OXR1*	41	0.572	0.7	34	0.876	1	34	0.779	1	41	0.365	0	34	0.497	1
*ATP2C2*	161	0.895	1	122	0.460	0	131	0.317	1	159	0.359	1	118	0.650	1
*SETBP1*	106	0.777	1	88	0.790	1	91	0.365	1	105	0.322	1	*85*	*0.018*	*1*
*CNTNAP2*	0	NA	NA	0	NA	NA	0	NA	NA	0	NA	NA	0	NA	NA

The number of variants refers to the number of variants passing QC and count/frequency thresholds for each gene. Associations in italic are only nominally significant

*ASD* autism spectrum disorder, *ADHD* attention deficit/hyperactivity disorder, *NA* not applicable

## Discussion

Our extended study replicated the results of our pilot study, namely, that, overall, there does not seem to be statistically significant genetic overlap between SLI and ASD or ADHD. However, the degree of overlap between SLI and ASD was determined more accurately in this study, and the new result was nominally significant, before correction for multiple testing (*P* = 0.037). Moreover, we observed a difference between the model for childhood autism and the model for Asperger’s syndrome in terms of the predictive ability of the PRS, suggesting some pleiotropy between childhood autism and SLI: while still explaining only a small proportion of the risk of childhood autism, the SLI-trained PRS nonetheless achieved statistical significance (surviving Bonferroni correction) only in the former case. The analyses in Table 1 for these two phenotypes test each case group against controls, which may be shared between the two case-control datasets and hence are not independent. When we test both case groups against each other, we find a significant difference, with a tendency for the childhood autism group to have a higher SLI-trained PRS. Keeping in mind that the PRS represented log-additive genetic risk of language impairment, this result shows an intriguing correspondence between this genetic difference between childhood autism and Asperger’s syndrome, which could be seen as a difference in the “genetic load for language impairment,” and the language profiles of the two disorders, which constitute the major difference between them [96]: ASD includes a group of pervasive developmental disorders involving abnormal social interaction, abnormal behavior patterns (typically involving restricted, stereotyped and repetitive behavior), and impaired communication [6]. Childhood autism is characterized by deficits in all of the above domains, while a diagnosis of Asperger’s syndrome is typically given when there are no evident communication deficits or language delay. In this study, the childhood autism and Asperger’s syndrome diagnoses followed the ICD guidelines (almost exclusively ICD-10 for the former, and only ICD-10 for the latter). The ICD-10 criteria for childhood autism specify, among other things: the characteristic type of abnormal functioning in all the three areas of psychopathology: reciprocal social interaction, communication, and restricted, stereotyped, repetitive behavior. For Asperger’s syndrome, it states: it differs from autism primarily in the fact that there is no general delay or retardation in language (https://icd.who.int/browse10/2019/en#/F84, accessed May 16, 2021). It should nonetheless be acknowledged that there may yet be some language problems associated with Asperger’s syndrome, too [97, 98], only not to the same extent as in childhood autism, and some theorize that childhood autism and Asperger’s syndrome are quantitatively different, rather than qualitatively different [96]. We also observed that a small number of children seemed to “transition” from one diagnosis to the other, or get both codes, although this could be the result of a misdiagnosis or some other kind of error inherent to registry-based research. In summary, the results of our PRS analyses indicate a subtle, but statistically significant, difference in the genetic load for language impairment between childhood autism and Asperger’s syndrome. This suggests that, at least at the group level, these two ASD subtypes can be distinguished by their genetic risk of language impairment, although the difference is very small, as reflected in the AUC.

In the rare variant analyses, three genes were nominally significantly associated with a disorder. Variants in *NDST4* were associated with ASD, variants in *RORB* were associated with childhood autism, and variants in *SETBP1* were associated with schizophrenia. *NDST4* was included due to its implication in language impairment [80]. The gene belongs to a family of genes called GlcNAcN-deacetylase/N-sulfotransferases, which have important roles in development [99]. While its connection to language is not clear, it has been associated with traits such as drinking behavior [100] and circulating levels of resistin, a hormone involved in inflammation [101], and some protein-truncating variants have been reported for this gene in the context of schizophrenia [102]. *RORB* was included due to its association with verbal intelligence, namely, with a vocabulary measure [82]. The protein encoded by this gene is a nuclear receptor [103] and it has been implicated in bipolar disorder [104]. Given its association with vocabulary, it is not surprising that it should show some association with childhood autism, as one study showed poor vocabulary growth to be associated with autism severity at 6 months from the start of the study (the participants’ initial chronological ages were 20–71 months) [105]. Notably, this gene has been highlighted in a recent ASD exome-sequencing study which included the iPSYCH sample but used a different methodology [106]. Lastly, *SETBP1* was included due to its implication in language impairment [79]. This gene is a transcription regulator [107], and it has been implicated in several studies of related disorders, such as childhood apraxia of speech [108] and developmental delay/expressive language delay [109–111]. There is some new evidence for its involvement in schizophrenia in a recent study [112], and it was also significant in some of the analyses of an exome-sequencing study of ASD [106].

### Limitations of our study

The training dataset for the PRS used in this study is, to the best of our knowledge, the only GWAS of SLI to date. As the primary sample was collected about 20 years ago and was originally intended for linkage analyses, it consists mainly of families of SLI probands, where unaffected individuals are related to affected individuals. The GWAS sample included several hundred individuals in subsets of case-parents trios, case-parent duos, cases, and so on, and, in the specific family-based GWAS design employed, only case subsets were used (i.e., controls were not used in the association tests themselves). As such, this analysis is inherently different from a standard case-control GWAS. While the SLI Consortium sample is not large by today’s standards of case-control studies, it is not atypical for family-based genetic studies. Another limitation is that the iPSYCH sample had no SLI phenotype or any kind of standardized language test score. However, the results from the pilot study sample that we obtained for our positive control (SLI) both in terms of the Nagelkerke’s *R*^2^ and the adjusted *R*^2^ were, in fact, higher (Table 1)^2^ than the maximum value obtained for schizophrenia (3.2%) in the study which conceptualized PRS analyses for human disease [37]. For schizophrenia, the *R*^2^ rose to 18.4% with a much larger discovery dataset (from a meta-analysis) a few years later [113], but a similar meta-analysis is currently not feasible for SLI. In summary, one limitation of our study is the sample size of the original GWAS, although it should be emphasized that we employed several tests and controls to assess whether the PRS predicts what it is supposed to predict, and we followed the conventional guidelines for PRS analyses in which the discovery sample was small, as explained in the “Methods” section. It should also be mentioned that, even though the *R*^2^ in the aforementioned original schizophrenia study was lower than in our study, the PRS it was based on was nonetheless used in a cross-disorder analysis, much like in this study.

A limitation in terms of the applicability of the results is that the difference in the SLI-trained PRS between childhood autism cases and Asperger’s syndrome cases was not large enough for clinical utility; the AUC for this model was too small for this at this stage, but, as proof of concept, our results are nonetheless promising; they suggest three things: (i) that, as observed in the pilot study, an SLI-based PRS is not a good predictor of ASD, meaning that the genetic correlation between the disorders is not expected to be large; (ii) that there is, however, a small but significant positive genetic overlap between SLI and childhood autism in particular, meaning that some loci could be shared between the two disorders (which could potentially be many loci with small effects)—these two results can inform us on the relationship between SLI and ASD and childhood autism; and (iii) that those overlapping loci could potentially distinguish between two types of autism spectrum disorder, one in which language is typically impaired, and another in which it is not. Given adequate training sets and sample sizes, this could, in the future, lead to a way of distinguishing between subtypes of ASD using genetic risk scores trained on language impairment. In the rare variant analyses, none of the tests survived a Bonferroni correction for multiple testing, and, therefore, they can provide at most suggestive evidence for association at this stage. This could be due to lack of power, as only a subset of the iPSYCH sample was exome-sequenced, and, by definition, rare variants are found in low numbers across samples. It should be noted that, when genetic correlation is not observed (i.e., even if it equals or is close to zero), it does not mean that pleiotropy does not exist, as the former depends on the directionality of the effects [2, 114]. In this context, it is worth noting that PRS cross-disorder analysis typically agrees with genetic correlation analysis [36]. It should also be noted that, while pleiotropy can give rise to genetic correlation, other factors could also influence an observed correlation, including misclassification of individuals into either disease group [115]. The SLI Consortium sample was examined for autism, and samples were excluded if they had an indication of autism; similarly, the ICD criteria require specific social and behavioral impairments for a diagnosis of childhood autism, which a child with SLI should not typically exhibit. However, it cannot be ruled out that a misclassification did occur.

## Conclusions

Our study did not find significant genetic overlaps between SLI and ASD, ADHD, and schizophrenia. However, a small but significant genetic overlap between SLI and childhood autism, in particular, was found. As this was not observed for Asperger’s syndrome, and the difference in PRS between the two case groups was significant, it may suggest that these two disorders, which differ linguistically, could also be distinguished genetically using polygenic risk scores for language impairment. While we found some overlaps across candidate genes for SLI and ASD, childhood autism, and schizophrenia, these associations did not survive Bonferroni correction and can, at most, provide suggestive evidence for pleiotropy. Taken together, our results may suggest that there is a number of loci that influence “pre-linguistic” mechanisms that influence neurodevelopment in general and thus may have an impact on language ability down the road, or loci that influence linguistic ability that is not domain-specific, which are shared between SLI and ASD, and, in particular, SLI and childhood autism. However, at this point, this is only speculative. Larger discovery samples may be needed in order to obtain a more reliable PRS, and larger exome-sequenced samples may be needed to detect the effects of rare variants, although our results may also suggest that rare variants in language-related genes may not have pleiotropic effects on the investigated neurodevelopmental disorders.

## Supplementary Information

**Additional file 1. Supplementary notes. **Furtherinformation about the EMIM models.

**Additional file 2: Supplementary Figure S1.** Flowchart for the rare variant analyses.

**Additional file 3: Supplementary Table S1.** Top 10 SNPs from the summary statistics from the SLI GWAS.

## Data Availability

The analyses performed in this study used summary statistics and datasets from previously published papers, as referenced in the text; no discovery analyses were performed. Data availability information for these datasets can be found in the relevant papers.

## Abbreviations

ADHD: Attention deficit/hyperactivity disorder
ASD: Autism spectrum disorder
AUC: Area under the curve
CI: Confidence interval
GWAS: Genome-wide association study
HLA: Human leukocyte antigen
MAF: Minor allele frequency
MHC: Major histocompatibility complex
OR: Odds ratio
PRS: Polygenic risk score
QC: Quality control
SD: standard deviation
SLI: Specific language impairment
SNP: Single-nucleotide polymorphism

## Footnotes

1 We use the term SLI in this paper, as the studies cited here are from before the change took place and used the more stringent definition and diagnostic criteria.

2 The values rise to Nagelkerke’s *R*^2^ = 3.87% and adjusted *R*^2^ = 6.05% (*P* = 0.024, with the updated dataset), when less stringent criteria are applied to SLI controls (i.e., when controls have language test scores above the threshold defining cases (1.5 SD below the population mean), as opposed to above a score defined as 0.5 SD below the population mean, as used in the main analyses), similar to what has been shown previously [49]).
