# Correction: Pleiotropy between language impairment and broader behavioral disorders—an investigation of both common and rare genetic variants

**DOI:** 10.1186/s11689-023-09499-5

**Published:** 2023-09-06

**Authors:** Ron Nudel, Vivek Appadurai, Alfonso Buil, Merete Nordentoft, Thomas Werge

**Affiliations:** 1 Institute of Biological Psychiatry, Mental Health Centre Sct. Hans, Mental Health Services Copenhagen, Roskilde, Denmark; 2 The Lundbeck Foundation Initiative for Integrative Psychiatric Research, iPSYCH, Aarhus, Denmark; 3 CORE - Copenhagen Research Centre for Mental Health, Mental Health Centre Copenhagen, Copenhagen University Hospital, Copenhagen, Denmark; 4 Department of Clinical Medicine, Faculty of Health and Medical Sciences, University of Copenhagen, Copenhagen, Denmark

**Correction: J Neurodevelop Disord 13, 54 (2021)**

10.1186/s11689-021-09403-z

Following the publication of the original article [1], we the authors have discovered two errors pertaining to the rare variant analyses in our article. Namely, one gene (*CNTNAP2*) was not tested in the original study, although it was selected to be tested; in the article, we noted that no variants passing our filters were found for the gene, but we now know that this was due to an error in the extraction of the gene regions from the larger dataset, where only this gene was not extracted. Additionally, it has come to our attention that the program used for the statistical testing in the rare variant analyses (EPACTS) did not handle multiallelic sites properly. Since our data had some multiallelic sites, this had an effect on the results of the tests. We have now re-extracted the gene regions (including the gene previously left out) and split each multiallelic site to biallelic sites with BCFtools v1.9, and performed the variant annotation and filtering based on variant effect type with snpEff v4.3t, as described in the original article (all gene transcripts for the genes of interest in the annotation reference (GRCh37.p13.RefSeq) were considered, and only sites for which the first gene mentioned in the annotation was on our list of candidate genes were used). The same statistical tests were then re-run with EPACTS v3.2.6 with the same parameters and on the same types of variants as described in the original article. The results do not change much: as before, no gene-phenotype association remains significant after Bonferroni correction. Two associations (*NDST4* and ASD; *RORB* and childhood autism) of the three that were nominally significant (*P*-value ≤ 0.05) in the original analyses remain so. No significant association between the added gene (*CNTNAP2*) and any phenotype was observed. When multiallelic sites are completely removed, the above two associations remain nominally significant, and all other ones are not significant (data not shown). Lastly, please also note that the study cited in the text as the “starting point” for the rare variant analyses describes the exome-sequencing and quality control, but the dataset has been updated with the addition of more samples. In summary, the conclusions of our study do not change. We have updated Table 3 to show the correct results.

**Table 3:** Results of the rare variant analyses

Phenotype	ASD			Childhood autism			Asperger’s syndrome			ADHD			Schizophrenia		
Gene	Number of variants	*P*-value	Rho	Number of variants	*P*-value	Rho	Number of variants	*P*-value	Rho	Number of variants	*P*-value	Rho	Number of variants	*P*-value	Rho
*NOP9*	63	0.067	0	47	0.165	0	50	0.103	0.5	57	0.233	0	47	0.115	0.3
*KMT2D*	465	0.250	0	355	0.670	0	368	0.545	1	437	0.737	1	337	0.136	1
*RORB*	14	0.243	1	*11*	*0.033*	*1*	12	0.894	1	14	0.300	1	11	0.396	1
*ABCC13*	1	0.138	NA	1	0.180	NA	1	0.584	NA	1	0.383	NA	1	0.079	NA
*NDST4*	*46*	*0.009*	*0.4*	43	0.189	1	44	0.124	0.4	49	0.238	1	45	0.148	1
*FAT3*	366	0.291	1	291	0.426	1	293	0.437	1	342	0.150	0.6	280	0.733	0
*CMIP*	22	0.402	1	18	1.000	1	18	0.637	0	23	1.000	0	17	0.715	0
*PALB2*	84	0.558	0	65	0.583	0	68	0.835	0	76	0.674	1	66	0.622	0
*SCN9A*	131	0.094	0	104	0.572	0	110	0.408	0	127	0.148	0	103	0.327	1
*MUC6*	32	0.911	1	28	0.810	0	29	1.000	1	33	0.843	1	29	0.864	1
*NFXL1*	26	0.519	1	13	0.695	0	15	0.235	1	17	0.849	1	13	0.559	0
*OXR1*	41	0.572	0.7	34	0.876	1	34	0.779	1	41	0.365	0	34	0.497	1
*ATP2C2*	167	0.880	1	120	0.448	0	132	0.288	1	161	0.226	1	116	0.821	1
*SETBP1*	109	0.850	1	90	0.884	1	92	0.593	1	109	1.000	1	86	0.380	1
*CNTNAP2*	122	0.527	0	96	0.280	0	104	0.763	1	120	0.235	0.9	93	0.840	0

The number of variants refers to the number of unique variants passing QC and count/frequency thresholds for each gene for each phenotype (this is not the variant count in individuals). Associations with summary statistics in italics were (only) nominally significant

*ASD* autism spectrum disorder, *ADHD* attention deficit/hyperactivity disorder, *NA* not applicable
