# Supplementary material for: Pleiotropy between language impairment and broader behavioral disorders—an investigation of both common and rare genetic variants
Source: J Neurodev Disord. 2021 Nov 13;13:54. doi: 10.1186/s11689-021-09403-z (PMC8590378; doi:10.1186/s11689-021-09403-z)
Supplement: Supplementary file 1 — Additional file 1. Supplementary notes. Furtherinformation about the EMIM models. [file 11689_2021_9403_MOESM1_ESM.pdf]

Supplementary notes for: Nudel *et al.* / Pleiotropy between language impairment and broader behavioral disorders – an investigation of both common and rare genetic variants: further information about the EMIM models

These notes are intended to provide further information about the model used in the discovery GWAS and its application in the generation of polygenic risk scores (PRS) in the current study. Examples throughout are based on the specific language impairment (SLI Consortium) sample as described in the original GWAS paper (1), but note that an updated version of the dataset was used, as described in our later publication (2), which was also used for the PRS analyses in the current paper.

The GWAS was performed using PREMIM (to prepare the input to EMIM) and EMIM (to run the model) (3). EMIM uses the models described in an earlier publication (4). PREMIM uses an algorithm to select the most informative family subset from every child-parent unit. For example, a case-parents trio is considered the most informative subset; when, for a given marker, both a case and their two parents have genotypes, then PREMIM will form a trio for this unit for the given marker. EMIM then uses information from across all family subsets for a given marker to estimate genotype relative risk parameters that increase (or decrease – by default the "risk" allele is the minor allele as detected by PREMIM, but it can also be associated with reduced risk, as reflected in risk parameters that are below 1) the probability that a child is affected. These parameters can then tested using a likelihood ratio test, whereby the null model has the parameter/s of interest fixed to 1 (these parameters are multiplicative), and the full or alternative model includes it/them as free parameter/s, as estimated from the data. Using the value of twice the difference between the maximized log-likelihoods of these nested models (a statistic which is supplied by EMIM) with the appropriate degrees of freedom (the difference in the number of

free parameters in the models) a p-value can be calculated using the chi-squared distribution as in a likelihood-ratio test.

The choice of risk parameters in the model

EMIM allows the choice of several risk parameters (and some combinations are also possible). For example, the top result in the original GWAS with the updated dataset (for rs4280164) was with a paternal parent-of-origin effect, whereby allele A had a paternal imprinting parameter I_p (a multiplicative factor by which the probability of disease is multiplied if the child receives a (paternal) copy of the risk allele from their father) = 0.255 (*i.e.* A is protective) with P = 2.918×10^{-8}. In this case, the null model has all risk parameters, including I_p, fixed to 1, and, in the full model, only I_p is freely estimated. The above p-value is from the likelihood-ratio test, with one degree of freedom. But we can also add a child risk parameter to both the null and full models. In this case, both the full model and the null model have a free R_1 parameter (the factor by which the disease risk is multiplied if the child has a single copy of the risk allele, and assuming that the risk from the child's having two risk alleles is $R_2 = (R_1)^2$). Note that it is also possible to specify a separate parameter for having two risk alleles *i.e.* that R_2 should not be assumed to be the square of R_1; this will be discussed in more detail below. Now the full model has freely estimated I_p and R_1, and the null model has only a freely estimated R_1, resulting in a likelihood ratio test with one degree of freedom. As this test remains significant ($P = 8.22\times10^{-8}$), we can say that the paternal parent-of-origin effect is still significant when allowing for a child effect to be included in the model as well. Note that the risk parameters are multiplicative and can be combined. Thus, if the baseline risk is denoted with α (the probability of disease when the child and both parents have no risk alleles), and a maternal risk parameter (the effect of the mother's genotype on child risk) is denoted with S_1 or S_2, based on the number of risk alleles the

mother has, (similar to R_1 and R_2 as defined above, but referring to the mother's genotype), then, if a paternal parent-of-origin effect, a maternal effect, and a child effect all operate together, the risk of a child who is homozygous for the risk allele and whose mother is also homozygous for the risk allele would be $\alpha R_2 S_2 I_p$. That is because, in addition to the baseline risk, the child also has the risk parameter for having two risk alleles, the risk parameter for having a mother with two risk alleles, and the risk parameter for having inherited a risk allele from the father. Note that assuming $R_2 = (R_1)^2$ or $S_2 = (S_1)^2$ means we test only one parameter instead of two, which affects the number of degrees of freedom, but this assumption might not be true for all cases. Thus, the above model could have 5 degrees of freedom (estimating R_1, R_2, S_1, S_2 and I_p in the full model and have them all fixed to 1 in the null model) or 3 degrees of freedom, if we make the above assumptions. Essentially, EMIM tests whether, based on the mating type of the parents and other data from the family subsets, the parameters of interest improve the model when they are freely estimated and not fixed to 1, or when more free parameters are included compared to the case of the null model (with nested models).

Parameterization for the SLI GWAS used as the discovery GWAS for the PRS

Traditionally, a PRS for binary traits is based on a case-control GWAS. If the weights used in the calculation of the PRS are odds ratios (ORs), then the PRS calculation entails transforming the weights to the log scale typically using the natural logarithm, so that a multiplicative OR becomes an additive logOR. Hence, a person carrying two risk/effect alleles for a given marker would get a weight of 2×logOR, where the OR is for the effect of that allele. The PRS is summed up across all included markers. In our SLI discovery GWAS, the full model included a freely estimated R_1 and the constraint $R_2 = (R_1)^2$. No other risk parameters were freely estimated in the full model. The null model had all risk parameters fixed to 1. This resembles a traditional case-

control GWAS in terms of the reported effects (even though only case subsets were used in the GWAS). The weights used were thus the R_1 values, treated as ORs in the calculation of the PRS.

References:

1. Nudel R, Simpson NH, Baird G, O'Hare A, Conti-Ramsden G, Bolton PF, et al. Genome-wide association analyses of child genotype effects and parent-of-origin effects in specific language impairment. Genes, brain, and behavior. 2014;13(4):418-29. Epub 2014/02/28.

2. Nudel R, Christiani CAJ, Ohland J, Uddin MJ, Hemager N, Ellersgaard DV, et al. Language deficits in specific language impairment, attention deficit/hyperactivity disorder, and autism spectrum disorder: An analysis of polygenic risk. Autism research : official journal of the International Society for Autism Research. 2020;13(3):369-81. Epub 2019/10/03.

3. Howey R, Cordell HJ. PREMIM and EMIM: tools for estimation of maternal, imprinting and interaction effects using multinomial modelling. BMC bioinformatics. 2012;13:149. Epub 2012/06/29.

4. Ainsworth HF, Unwin J, Jamison DL, Cordell HJ. Investigation of maternal effects, maternal-fetal interactions and parent-of-origin effects (imprinting), using mothers and their offspring. Genetic epidemiology. 2011;35(1):19-45. Epub 2010/12/25.
