# Supplementary material for: Pleiotropy between language impairment and broader behavioral disorders—an investigation of both common and rare genetic variants
Source: J Neurodev Disord. 2021 Nov 13;13:54. doi: 10.1186/s11689-021-09403-z (PMC8590378; doi:10.1186/s11689-021-09403-z)
Supplement: Supplementary file 2 — Additional file 2: Supplementary Figure S1. Flowchart for the rare variant analyses. [file 11689_2021_9403_MOESM2_ESM.pdf]

Supplementary figure for: Nudel *et al.* / Pleiotropy between language impairment and broader behavioral disorders – an investigation of both common and rare genetic variants.

Supplementary Figure S1: Flowchart for the rare variant analyses

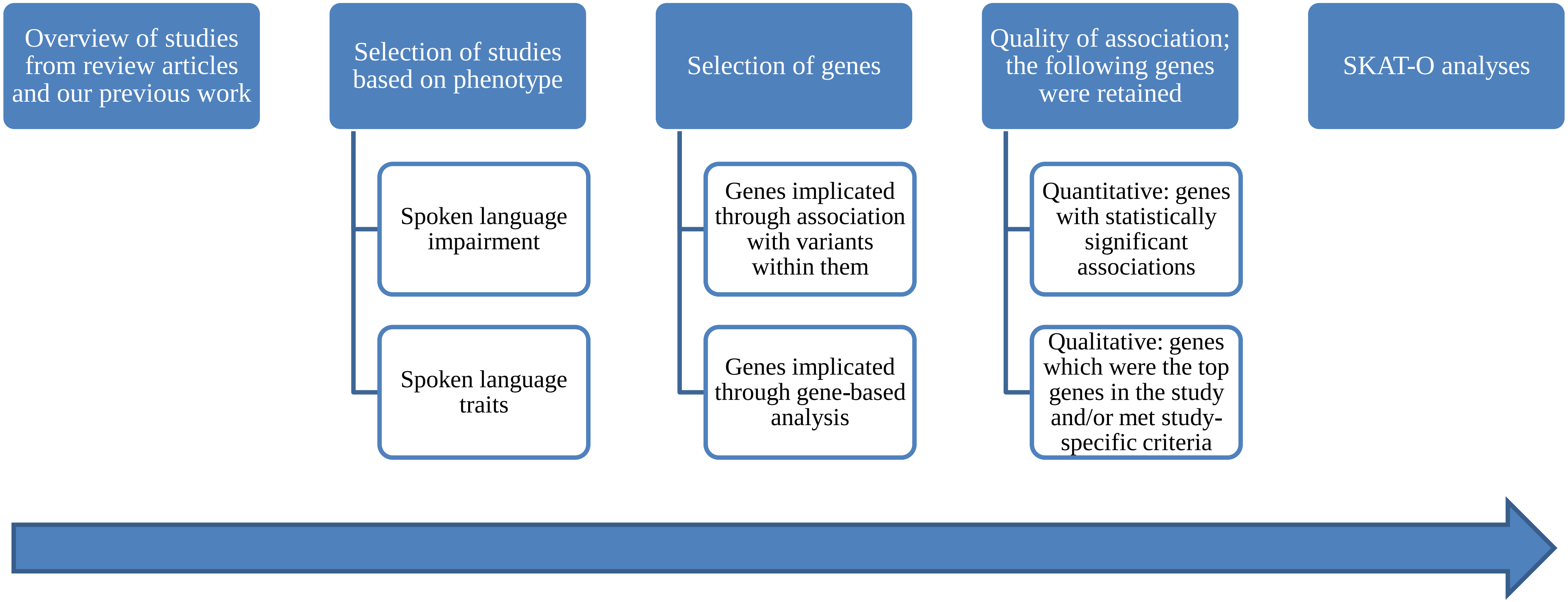

Mountford *et al.* (Table 1): 22 genes.
Guerra *et al.* (Table 1): 83 genes.
One gene not included in those tables was mentioned in our previous publication and was added to the list.

- 13 genes meeting all criteria implicated in spoken language disorders (12 harbored rare variants meeting our criteria in our sample).
- 2 genes meeting all criteria implicated in spoken language traits.
