# Supplementary material for: Pleiotropy between language impairment and broader behavioral disorders—an investigation of both common and rare genetic variants
Source: J Neurodev Disord. 2021 Nov 13;13:54. doi: 10.1186/s11689-021-09403-z (PMC8590378; doi:10.1186/s11689-021-09403-z)
Supplement: Supplementary file 3 — Additional file 3: Supplementary Table S1. Top 10 SNPs from the summary statistics from the SLI GWAS. [file 11689_2021_9403_MOESM3_ESM.pdf]

Supplementary table for: Nudel *et al.* / Pleiotropy between language impairment and broader behavioral disorders – an investigation of both common and rare genetic variants

Supplementary Table S1: Top 10 SNPs from the summary statistics from the SLI GWAS.

SNP	A_1	A_2	R_1 (relative to A_1)	P-value	Gene
rs7109365	G	A	2.417926	1.79×10^{-6}	*CNTN5*
rs4790018	G	A	1.974924	2.17×10^{-6}	*RBFOX3*
rs11876129	G	A	0.290492	3.98×10^{-6}	
rs574715	G	A	1.885985	9.32×10^{-6}	
rs7760531	G	A	0.527788	1.09×10^{-5}	*MDGA1*
rs4266409	A	G	0.23974	1.37×10^{-5}	
rs671986	G	A	1.86278	1.37×10^{-5}	
rs9874037	A	G	0.363081	1.42×10^{-5}	*THRB*
rs4977291	A	C	2.856994	1.52×10^{-5}	*DENND4C*
rs6452234	A	G	0.467306	1.77×10^{-5}	
